# Effect of Proprietary Chinese Medicine on Coronary Microvascular Dysfunction in Patients with Microvascular Angina: A Systematic Review and Meta-Analysis

**DOI:** 10.1155/2023/9242752

**Published:** 2023-01-24

**Authors:** Qiuyu Yu, Xiaoyu Xu, Shun Wang, Yu Fan, Jian Zhang, Yingshu Leng, Fuming Liu

**Affiliations:** ^1^Affiliated Hospital of Nanjing University of Chinese Medicine, Jiangsu Province Hospital of Chinese Medicine, First Clinical Medical College, Nanjing University of Chinese Medicine, Nanjing, Jiangsu, China; ^2^Department of Biomedical Engineering, University of Ottawa, Ottawa, Ontario K1N 6N5, Canada

## Abstract

**Background:**

Microvascular angina (MVA) has received increasing attention and interest in recent years, but there are still some shortcomings in the diagnosis and treatments at current stage. In recent years, several studies have confirmed the efficacy of proprietary Chinese medicines (PCMs) in improving MVA symptoms; however, there is no systematic review and meta-analysis to comprehensively assess the efficacy of PCMs in this area.

**Objective:**

Investigating the clinical efficacy of proprietary Chinese medicines for treating MVA and coronary microvascular function.

**Methods:**

We looked up articles from January 1, 2012, to the present from eight databases. Then, we screened the literature and followed the 2019 version 2 of Cochrane risk of bias tool for systematic review. The Stata/SE 15.0 software was used for the meta-analysis.

**Results:**

There are 21 studies, including 1,641 patients who were included in this review. According to the results, the combination of PCMs and conventional MVA treatment was able to further enhance clinical efficacy [RR = 1.28, 95% CI (1.20, 1.36), *p* < 0.001], prolong the time of duration on the treadmill exercise testing (TET) [SMD = 1.49, 95% CI (0.63, 2.36), *p* = 0.001] and improve levels of NO [SMD = −1.77 95% CI (−2.11, −1.43), *p* < 0.001]. At the same time, PCMs could also decrease the microvascular resistance index (IMR) [SMD = −1.79, 95% CI (−2.58, −1.00), *p* < 0.001)], serum level of hs-CRP [SMD = −1.21, 95% CI (−1.84, −0.58), *p* < 0.001] and ET-1 [SMD = −1.77 95% CI (−2.11, −1.43), *p* < 0.001]. Regards to medication safety, a total of 27 adverse events occurred, including 10 cases in the intervention group and 17 cases in the control group.

**Conclusion:**

The study suggests that the combination of PCMs and conventional MVA treatment enhances clinical efficacy and could better improve coronary microvascular function. In the future, we expect more high-quality, randomized, double-blind clinical studies to validate the safety, and efficacy of PCMs to provide valuable evidence-based medicine (EBM) for the treatment of MVA with PCMs.

## 1. Introduction

Chest pain is one of the most common clinical symptoms and is often caused by myocardial ischemia. However, not all ischemic angina can be detected by coronary angiography (CAG) for obstructive coronary artery stenosis. There is a study which shows that up to 70% of patients with angina do not have obstructive coronary stenosis during CAG [1]. Accordingly, we classified ischemic nonobstructive coronary artery disease (INOCA) as those with clinically relevant symptoms of ischemia, whereas no significant obstructive coronary stenosis (<50% stenosis) by coronary angiography. There is a possibility that patients with INOCA will miss the timing for treatment because they will be diagnosed with cardiogenic chest pain due to negative coronary angiography results. In this case, these patients will suffer from recurrent angina symptoms, which greatly affect their quality of life and predispose them to a sharp increase in the incidence of major adverse cardiovascular events (MACE) such as myocardial infarction and heart failure in the long term [2]. This is the reason cardiogenic chest pain caused by INOCA should be given adequate concern and appropriate therapeutic measures in a timely manner.

According to its pathophysiological characteristics, INOCA can be divided into two types: epicardial vasospastic angina (EVA) and microvascular angina (MVA). In this study, however, we focus on microvascular angina, the occurrence of which is closely related to coronary microcirculation dysfunction (CMD) [3]. As atherosclerosis, hypertension, diabetes, and other cardiovascular disease risks deteriorate, endothelial dysfunction and restriction of vasodilation increase, and the blood flow through the coronary microcirculation decreases. Subsequently, the coronary microcirculation becomes disturbed, leading to microvascular angina pectoris [4, 5].

The following tests are generally used as clinical references for the diagnosis of microvascular angina [6, 7]: (1) the index of microvascular resistance (IMR) is the ratio of distal coronary pressure to blood flow during the state of maximum congestion. (2) The treadmill exercise test, which monitors the duration of exercise and the maximum degree of ST-segment depression, evaluates the degree of myocardial ischemia under exercise load. In addition, serum inflammatory markers such as hypersensitive c-reactive protein and protein expressions such as endothelin-1 and nitric oxide, which reflect endothelial function, as well as the improvement in clinical symptoms can also be used to help physicians determine the severity and prognosis of the disease.

Currently, the management strategy of INOCA focuses on antianginal drugs, statins, ACEI/ARB, antiplatelet drugs, promicrovascular dilating drugs (e.g., ranolazine, etc.), and the control of risk factors (e.g., diabetes, hypertension, etc.) [8, 9]. The long-history developed Chinese medicine has been the health safeguard to Chinese for thousands of years. In traditional Chinese medicine (TCM), angina pectoris is classified as “chest impediment and heart pain,” whose basic pathogenesis is “yang deficiency and yin exuberance leading to internal cold.” The therapeutic principles focus on warming yang and replenishing qi, dissipating cold, resolving phlegm, activating blood, and providing a variety of treatment options [10]. The term “proprietary Chinese medicine (PCM)” refers to Chinese herbal medicine as raw materials processed by modern technology, with the advantages of quantitative dose, portability, convenient storage, etc [11]. In recent years, we have seen an increasing number of experimental studies on the efficacy of PCMs on MVA, exploring and affirming the clinical efficacy of PCM [12, 13]. The aim of this article is to investigate the clinical efficacy of PCM for treating MVA through a systematic review of the literature and meta-analysis to provide a more evidence-based basis for the treatment of MVA with PCMs.

## 2. Materials and Methods

### 2.1. Protocol and Registration

This systematic review and meta-analysis adhered to the Preferred Reporting Items for Systematic Reviews and Meta-Analyses protocol (PRISMA-P) and is registered in PROSPERO (registration number: CRD42022322261).

### 2.2. Search Strategy

We searched the following databases for publications in the past decade: PubMed, Embase, Cochrane Library, Web of Science, CNKI (China National Knowledge Infrastructure), VIP, Wan Fang, and CBM (Chinese Biomedical Database). Search terms included “traditional Chinese medicine,” “proprietary Chinese medicine,” “Chinese patent medicine,” “nonobstructive coronary arteries,” “Microvascular Angina,” “coronary microvascular,” “coronary artery microvascular,” “coronary artery microvasculature,” “coronary capillary,” “coronary artery capillary,” “coronary microcirculation,” “coronary artery microcirculation,” “microvascular Angina,” “coronary slow flow,” and “X syndrome.” We employed a blend of key words and free words in our search strategy. Certainly, to guarantee the integrity of literature retrieval, we also manually searched the aforementioned database for additional relevant published papers. Taking PubMed as an example, the detailed search strategy is shown in Table 1.

### 2.3. Inclusion Criteria

All of the included studies met the following criteria: (1) Each study was a randomized controlled trial (RCT) investigating the clinical efficacy of PCMs in the treatment of MVA; (2) Subjects were patients that clinically identified nonobstructive coronary ischemic disease (no obstructive stenosis or stenosis <50% on CAG) and without percutaneous coronary intervention (PCI) before; (3) the therapeutic drugs selected for the experimental group included oral PCMs, with exclusion of nonoral drugs; (4) the control group was treated with but not restricted to antiplatelet agents, statins, ACEI/ARB, coronary artery vasodilators, *β*-blockers, CCB, etc.; (5) the observation indicators in the study must cover at least one of the following: (a) index of microvascular resistance (IMR); (b) time of duration on the treadmill exercise testing (TET); (c) hypersensitive c-reactive protein (hs-CRP); (d) serum endothelin-1 (ET-1); (e) nitric oxide (NO).

### 2.4. Exclusion Criteria

Studies will be excluded if the following criteria are met: (1) non-RCT research, such as dissertations, conference papers, animal experiments, reviews, theoretical discussions, empirical summaries, etc.; (2) incomplete data or inaccessible full text; (3) the PCM in the research is for nonoral use.

### 2.5. Data Extraction

Two researchers independently reviewed the literature and extracted data based on inclusion and exclusion criteria. They cross-checked and discussed the retrieved data. If disagreement arose, they referred to the opinions of a third researcher to settle their discrepancies. Included in the extracted data were the first author's name, publication date, sample size, gender distribution ratio, mean age, intervention measures, treatment duration, outcomes, and adverse events.

### 2.6. Quality Evaluation

Two investigators independently assessed the quality of the included literature using the 2019 version 2 of the Cochrane risk of bias tool (RoB2), with the following risk of bias evaluation entries: (1) bias arising from the randomization process; (2) bias due to deviations from intended interventions; (3) bias resulting from missing outcome data; (4) bias in measurement of the outcome; (5) bias in the selection of the reported results. At last, a global risk of bias judgment was generated. The risk of bias assessment table established three evaluation levels: “low,” “high,” and “some concerns.”

### 2.7. Data Analysis

By using Stata/SE 15.0 software, we combined the dichotomous variables retrieved from the data using relative risk (RR). For the continuous variables in the extracted data, either weighted mean difference (WMD) or standardized mean difference (SMD) was utilized as the combined statistic in a random-effects model or a fixed-effects model. All effect measures were provided with a 95% confidence interval (CI). If there was no statistical heterogeneity among the studies (*p* > 0.1, *I*^2^ < 50%), a fixed-effects model was applied for the meta-combined analysis. If there was statistical heterogeneity among the studies (*p* < 0.1, *I*^2^ ≥ 50%), subgroup analysis was used to eliminate heterogeneity according to possible heterogeneity factors, and sensitivity analysis was performed as needed. Finally, forest plots were presented to demonstrate the overall results. Statistical significance was determined at *p* < 0.05. The publication bias was assessed by Egger's test.

## 3. Results

### 3.1. Search Results

**Figure 1:**
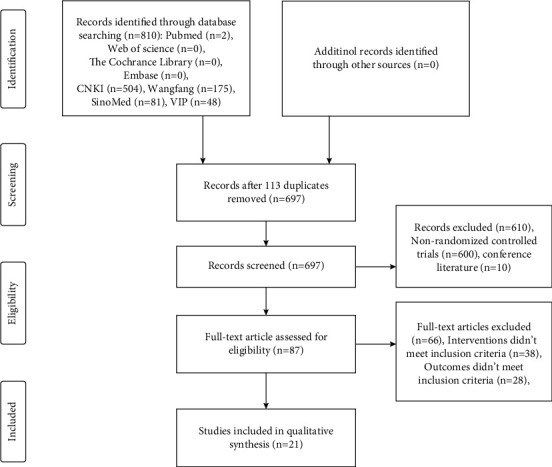
Flow diagram of literature screening.

From all databases, a total of 810 articles were retrieved, and subsequently, 113 duplicates were removed using EndNote software. After reviewing the titles and abstracts of the papers, we excluded 600 nonclinical RCT articles and 10 conference materials. Afterwards, 21 studies were obtained by screening for systematic review and meta-analysis, and 66 articles that did not fulfill the inclusion criteria (*n* = 38) or outcomes that did not meet the inclusion criteria (*n* = 28) were excluded. The comprehensive screening procedure is shown in Figure 1.

### 3.2. Study Characteristics

These 21 studies comprised a total of 1,641 patients (826 in the intervention group and 815 in the control group). Detailed descriptions of the characteristics of these studies are presented in Table 2.

### 3.3. Literature Bias and Quality Assessment

**Figure 2:**
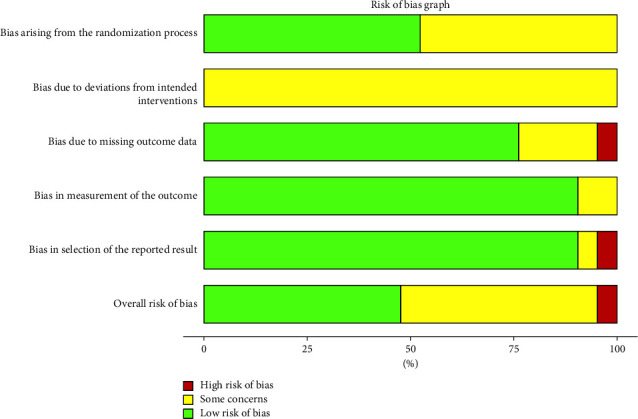
Risk of bias graph.

**Figure 3:**
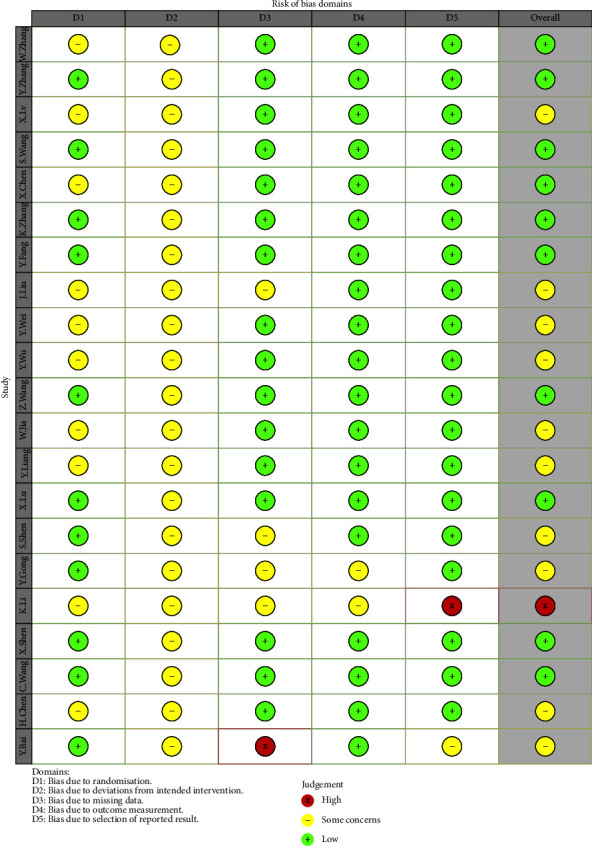
Risk of bias summary.

Eleven articles [14, 17, 19, 20, 24, 27–29, 32, 34] discussed the use of the random number table method for grouping, while the remaining ten articles [14, 16, 18, 21, 22, 25, 26, 30, 33] discussed random assignment without specifying the method. In one research study [16], six participants dropped out without explanation. All twenty-one studies gave data for all observed indicators of the intended assay, and no other bias was identified. The Cochrane bias risk results are shown in Figures 2 and 3.

### 3.4. Efficacy Assessment

#### 3.4.1. Clinical Efficacy of PCMs (The Improvement of Angina)

**Figure 4:**
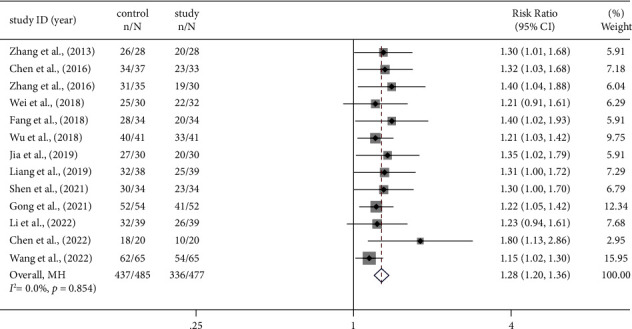
Forest plot of clinical efficacy.

A total of thirteen studies [14, 18–20, 22, 23, 25, 26, 28–30, 32] reported the overall clinical effectiveness of PCMs treatment based on the improvement of angina as an outcome criterion. A meta-analysis revealed that there was satisfactory homogeneity among these 13 studies. (*I*^2^ = 0.0%, *p* = 0.854) (Figure 4). Thus, we applied a Mantel–Haenszel model to analyze the combined data from these studies. The results showed that PCMs combined with conventional MVA treatment could further improve the clinical symptoms [RR = 1.28, 95% CI (1.20, 1.36), *p* < 0.001] (Figure 4).

#### 3.4.2. Index of Microvascular Resistance (IMR)

**Figure 5:**
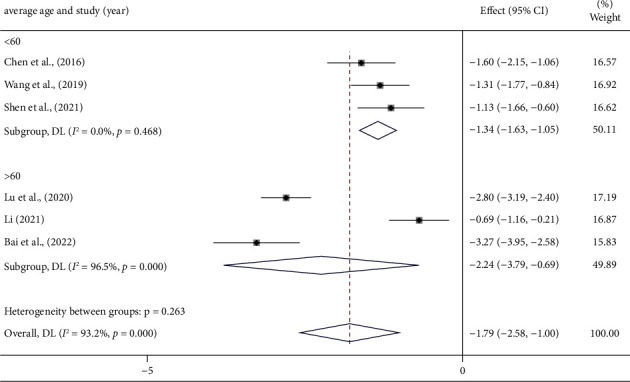
Forest plot of IMR and subgroup analysis based on average age.

IMR results have been reported in six studies [18, 24, 27, 28, 30, 34]. Since meta-analysis revealed high heterogeneity (*I*^2^ = 93.2%, *p* < 0.001) (Figure S1), we performed subgroup analysis for potential factors contributing to high heterogeneity. The outcomes suggest that the average age of the participants may be a source of heterogeneity (Figure 5). Notably, there was still considerably high heterogeneity in the remaining subgroups (Table 3) (Figures S2–S6), suggesting that additional factors may have contributed to the heterogeneity.

Taking all aspects into account, we suspect that the source of heterogeneity may be related to the multiple variables that differ from operator to operator as well as provider to provider in terms of testing methods and evaluation criteria. Thus, the random-effects model was used to examine the data of these six trials. The results suggested that the PCM intervention could further decrease the IMR values. (SMD = −1.79, 95% CI (−2.58, −1.00), *p* < 0.001)) (Figure 5).

#### 3.4.3. Time of Duration on the Treadmill Exercise Testing (s)

**Figure 6:**
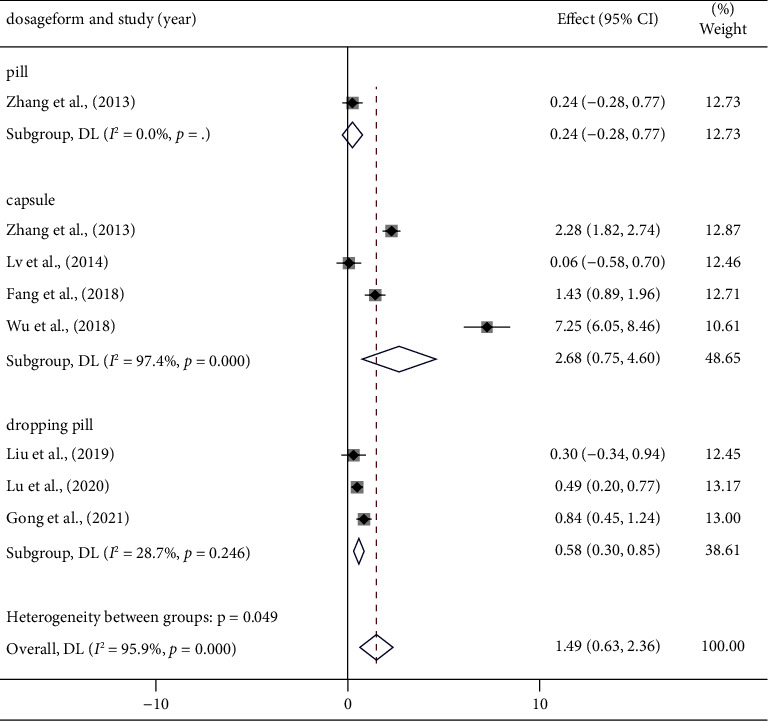
Forest plot of TET and subgroup analysis based on dosage form.

Eight articles [14, 16, 20, 21, 23, 27, 29] reported the findings of the duration on the treadmill exercise testing. We discovered there is high heterogeneity in the results of these eight studies (*I*^2^ = 95.9%, *p* < 0.001) (Figure 6) (Figure S7). As a result, we performed a subgroup analysis of the meta-analysis results (Table 4) (Figures S8–S13), indicating that heterogeneity was significantly reduced in some experimental groups, including dosage form, drug, and the average. To better explore the relationship between these factors and heterogeneity, we conducted a regression analysis on these eight studies; however, the relationship was not as strong as expected (the regression analysis was unable to include “drug” factors due to an insufficient number of studies) (Table S1).

TET detection can vary methodologically between providers for the same reasons as in the previous article. And even though we found that heterogeneity was eliminated in the STDP drug group, the effect of drug composition on heterogeneity could not be excluded due to the insufficient number of included literature studies. Finally, we applied a random-effects model to the meta-analysis, and the results showed that the combination of PCMs and conventional MVA treatment was able to prolong time on the treadmill exercise testing. [SMD = 1.49, 95% CI (0.63, 2.36), *p* = 0.001].

#### 3.4.4. Hypersensitive C-Reactive Protein (mg/L)

Nine articles [14, 17, 19, 21, 24–26, 29, 33] published hs-CRP data. As the findings of the meta-analysis indicated significant heterogeneity (*I*^2^ = 92.6%, *p* < 0.001) (Figure 6) (Figure S14), we further ran a subgroup analysis to determine the causes of high heterogeneity (Table 5) (Figures S15–S21). Owing to the large number of publication year groups, only those years with a significant decrease in heterogeneity were listed in Table 4, and the same applied to the drug group. The meta-regression analysis results can be found in the Supplementary Material (for the same reason as mentioned above, the regression analysis did not include “drug”) and the results showed little association between the subgroups with high heterogeneity (Table S2).

**Figure 7:**
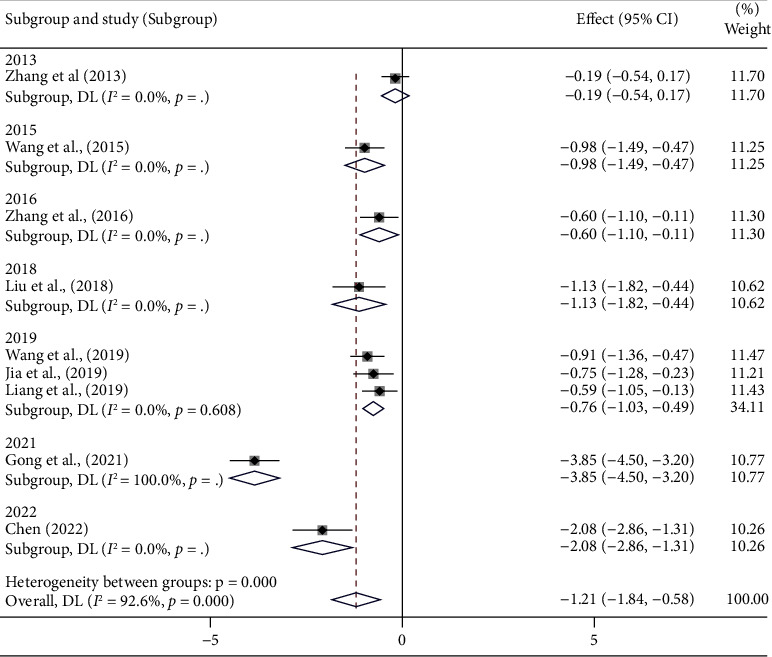
Forest plot of hs-CRP and subgroup analysis based on year.

Since some heterogeneity still cannot be eliminated, we believe that differences in testing equipment and operational methods may be responsible for the high heterogeneity, whereas these differences are acceptable, so we used random-effects model to analyze the results, which demonstrated that the reduction of hs-CRP in patients treated with PCMs was more significant. [SMD = −1.21, 95% CI (−1.84, −0.58), *p* < 0.001] (Figure 7).

#### 3.4.5. Endothelin-1 (ET-1) (ng/L)

**Figure 8:**
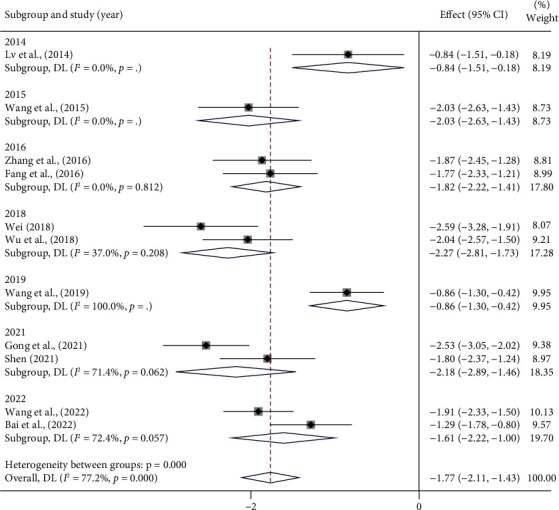
Forest plot of ET-1 and subgroup analysis based on year.

A total of eleven studies [16, 17, 19, 20, 22–24, 29, 31, 32, 34] reported the results for ET-1. The meta-analysis revealed high heterogeneity (*I*^2^ = 77.2%, *p* < 0.001) (Figure 8) (Figure S22), so we repeated subgroup analysis (Table 6) (Figures S23–S29). Similarly, Table 6 shows only those publication years with a significant decrease in heterogeneity and those drugs with sufficient literature for subgroup analysis (Table 6).

The high heterogeneity in the remaining subgroups cannot be overlooked, and the regression analysis results showed that the occurrence of high heterogeneity could not be fully explained by these subgroups mentioned above other than drugs (Table S3). For the same considerations as hs-CRP, factors such as different brands of kits and different personnel using different assays when performing serological index tests could be the potential triggers for the high heterogeneity in the above results. And the differences in drug composition certainly cannot be ignored. We combined the results of the studies using a random-effects model, which suggested that ET-1 was significantly lower in patients treated with the appropriate combination of PCMs compared with conventional MVA treatment alone. [SMD = −1.77 95% CI (−2.11, −1.43), *p* < 0.001] (Figure 8).

#### 3.4.6. Nitric Oxide (NO) (*μ*mol/L)

Eleven studies [16, 19, 20, 22–25, 29, 31, 32, 34] reported the results for NO. Meta-analysis showed high heterogeneity (*I*^2^ = 80.0%, *p* < 0.001) (Figure 9) (Figure S30), and then we conducted subgroup analysis (Table 7) (Figures S31–S37). For the same reasons as above, Table 7 lists only some of the years and the drug subgroups.

**Figure 9:**
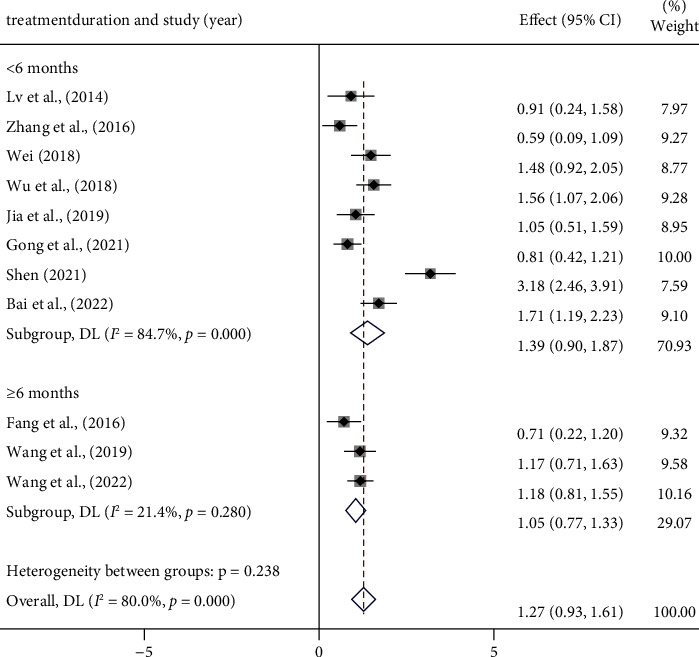
Forest plot of NO and subgroup analysis based on treatment duration.

Interestingly, the regression analysis results indicate that the dosage form is likely to be the cause of heterogeneity (*p* < 0.05) (Table S4), which is not consistent with the results of the subgroup analysis (Table 7). We believe that this is related to the small number of pieces of literature included. What's more, the high heterogeneity may also be related to differences in test manipulation, kit, and assay equipment, as well as drug composition in different treatment groups. We finally used a random-effects model for the overall effect amount analysis, and the results showed that PCMs can effectively increase serum NO levels in MVA patients [SMD = −1.77 95% CI (−2.11, −1.43), *p* < 0.001] (Figure 9).

#### 3.4.7. Safety

In none of the studies were major adverse responses or malignant cardiovascular events attributed to PCMs reported. Four studies [14, 22, 31, 32] mentioned treatment-emergent adverse reactions, and five studies [14, 18, 23, 27, 33] stated unequivocally that no significant adverse effects were observed during the experiment, leaving 12 studies that did not mention adverse effects of drug treatment (Table 8).

From the foregoing adverse reaction statistics, it is evident that the treatment of MVA with PCMs is safer, and the adverse reactions induced are generally minor and infrequent. Due to the inadequacy of the total sample size and the existence in some studies of a greater number of adverse reactions in the intervention group; however, we still need to be cautious about the outcome of the drug safety analysis. Nevertheless, it is encouraging to see that in recent years, a growing number of studies have shown the clinical safety of proprietary Chinese medicines [35, 36].

#### 3.4.8. Publication Bias

We utilized the Egger test module of Stata 15.0 to identify publication bias for all the indicators. The outcomes are presented in Table 9, where we can find that most of the indicators are free of publication bias (*p* > 0.05), except for “hypersensitive C-reactive protein” (Table 9). Therefore, we should be cautious about the conclusions of the studies regarding this indicator.

## 4. Discussion

This meta-analysis comprised 1,641 patients from 21 trials (826 in the intervention group and 815 in the control group). Compared to the conventional therapy of MVA, the addition of PCMs could improve angina symptoms, reduce microvascular resistance, prolong the total duration of the treadmill exercise test, decrease the serum levels of hs-CRP and ET-1, as well as increase the levels of NO. Therefore, we concluded that PCM combined with conventional treatment can enhance coronary microcirculation and improve vascular endothelial function in patients with MVA.

IMR, an intracoronary guidewire-based technique, is a crucial indicator that can reflect coronary circulatory function. When compared to other similar indicators, it is less affected by coronary hemodynamics, and provides a more accurate and quantitative assessment of CMD [37, 38]. In recent research, it has become the preferred endpoint for evaluating CMD treatment strategies. Clinical studies have demonstrated that IMR aids in predicting and grading the severity of microvascular obstruction [37]. After combined analysis in a random-effects model, the combination of PCMs and the conventional MVA treatment group was able to further reduce IMR compared to the control group [SMD = −1.79, 95% CI (−2.58, −1.00), *p* < 0.001)]. Since there was high heterogeneity (*I*^2^ = 93.2%, *p* < 0.001), we ran a subgroup analysis of the results and found that in the subgroup of participants with an average age less than 60 years, heterogeneity was much lower (*I*^2^ = 0.00%, *p* = 0.468) (Figure 5). This indicates that age variables are strongly associated with IMR. Notably, subgroup analysis for dosage forms showed that the reduction of IMR by dropping pills was not statistically different (*p* > 0.05), suggesting that the pill form may be more suitable for reducing IMR in MVA patients (Table 3). Meanwhile, it cannot be ignored that the heterogeneity was still high in the other subgroups, so we further performed a regression analysis but found nothing strongly associated with the high heterogeneity. Different ingredients in PCMs and different medical institutions employing different procedures for IMR testing are the potential causes of the significant heterogeneity. Regardless of this, it is difficult to eliminate these variances due to the nature of clinical trials.

An increase in exercise tolerance is important for the enhancement of coronary microcirculation function. In order to evaluate the efficiency of targeted therapy for MVA, the duration of treadmill exercise testing (TET) is frequently employed. According to clinical studies, treadmill exercise testing results can predict all-cause mortality in adults who have coronary artery disease with normal electrocardiograms [38]. In the current study, eight articles reported the results of the TET, and combined calculations based on a random-effects model evidenced that the PCM intervention group was able to further prolong the total duration of the treadmill exercise testing in MVA patients and effectively improve exercise tolerance in subjects [SMD = 1.49, 95% CI (0.63, 2.36), *p* = 0.001] (Figure 5). Due to the substantial heterogeneity, we did subgroup analysis, and the results indicated that the drug itself, the dosage form of PCMs, and the average age of the participants were associated with heterogeneity. Though, the heterogeneity could not be minimized in the remaining subgroups (Table 4), we suggest that the heterogeneity may be attributed to changes in testing equipment between medical institutions and the operators themselves.

The endothelial-dependent vasodilation and contraction dysfunction can result in myocardial ischemia and hypoxia, provoking angina pectoris, which is one of the primary causes of MVA [9, 41]. The inflammatory response is closely related to vascular endothelial function, which can inhibit microvascular dilation and lead to coronary microvascular dysfunction [5]. The hs-CRP is extensively used in clinical practice as a sensitive indicator of the degree of an inflammatory response to assess the risk of cardiovascular disease. In recent years, it has been shown that hs-CRP can be used as an independent serological marker and predictor of abnormal coronary artery responsiveness in patients with nonobstructive coronary artery disease [42]. In this article, nine papers reported results for hs-CRP, which were combined using a random-effects model to demonstrate that the treatment group with a combined PCM cointervention was able to further reduce serum levels of hs-CRP in patients with INOCA compared with the conventional INOCA treatment group [SMD = −1.21, 95% CI (−1.84, −0.58), *p* < 0.001]. Then, we repeated subgroup analysis and found that the heterogeneity was related to the drugs, dosage form, the gender ratio of the participants, and the publishing year (Figure 6). Moreover, in the subgroup with a sample size greater than 80 and an average age greater than 60, we found no statistically significant differences among the conclusions of the studies. This seems to indicate to us that PCM is not effective in improving hs-CRP in elderly patients. Since the results of the regression analysis indicate that the factors listed above are not the primary cause of high heterogeneity, we believe that the different testing equipment and testing methodologies applied by different medical institutions could be responsible for it.

Endothelin-1 (ET-1) and nitric oxide (NO) are both endogenous vasoconstrictors. On the contrary, ET-1 concentration is negatively correlated with coronary blood flow response in patients with CMD, while NO concentration is positively correlated [43, 44]. A recent study concluded that oral endothelin A receptor antagonists inhibited ET-1 contraction of blood vessels to relieve microvascular angina, suggesting that ET-1 is an important therapeutic target in coronary microvascular dysfunction [45]. Based on the random-effects model analysis of eleven articles reporting ET-1 results, we concluded that the combination of PCM with the conventional MVA treatment was more effective in reducing serum ET-1 levels and improving vascular endothelial function. However, considering the results of the high heterogeneity (*I*^2^ = 77.2%, *p* < 0.001), we performed a subgroup analysis. The results shown in Table 6 indicate that different publication years have an effect on heterogeneity. Then we look for NO, a serological marker closely related to vascular endothelial function. The reduction in nitric oxide bioavailability, leading to impaired endothelium-dependent vasodilatory function, is one of the important pathogenic mechanisms for MVA [46]. Our study shows that PCMs can effectively increase serum NO levels in MVA patients, which indirectly confirms the protective effects of PCMs on the vascular endothelium [SMD = −1.77 95% CI (−2.11, −1.43), *p* < 0.001]. And for the same reasons as for hs-CRP, we concluded that variances in assay equipment, assay procedures, and kits, as well as differences in ingredients, were related to the high heterogeneity.

This study is the first systematic review and meta-analysis to reveal the efficacy of PCMs in the treatment of microvascular angina by improving coronary microvascular function. The previous systematic reviews and meta-analysis have focused on the association between PCMs and coronary microcirculation, although the study population did not focus on patients with MVA [47, 48]. Secondly, the outcome evaluation indicators set in this study incorporated clinical symptoms, coronary microcirculation evaluation indicators (IMR), clinical symptom evaluation indicators (TET), and vascular function evaluation indicators (hs-CRP, ET-1, NO) for a more comprehensive and objective reflection of the efficacy of PCM in the MVA treatment. The 2021 guidelines for the evaluation and diagnosis of chest pain published by the American Heart Association emphasize the importance of diagnosis and treatment of non-obstructive coronary artery disease [49]. Due to negative coronary angiography results, patients will take the risk of missing the timing of treatment, leading to adverse cardiovascular events. Therefore, the need of accurate diagnosis and prompt treatment of INOCA is constantly increasing [9]. Current clinical treatment of MVA is focuses mostly on coronary artery dilation, coronary blood flow enhancement, cholesterol reduction and plaque stability, as well as antiplatelet aggregation [50]. The formulation of PCMs is based on the theory of Chinese medicine and incorporates modern pharmaceutical technologies [11]. Although there were differences among the eight Chinese patent medicines involved in this study, their drug effects were all centered on the basic treatment of “benefitting qi and warming yang, activating blood and resolving phlegm.”

Recent research has shown that PCMs can improve coronary microvascular dysfunction in rats by activating Nrf2 to inhibit vascular inflammatory response, and improve endothelial cell function and exert cardiovascular protective effects by inducing KLF5 expression in microvascular endothelial cells and increasing tight junction protein levels [51, 52]. This suggests that PCM has great therapeutic potential for improving coronary microcirculation, meanwhile more clinical trials and basic studies are necessary to verify this.

### 4.1. Limitation

Firstly, only 21 publications matched the inclusion criteria for the current study, which is a small amount. This is mainly due to the fact that there are fewer clinical studies in this area or that the observed indexes do not reflect the improvement of coronary microcirculation. Secondly, IMR, as the main indicator for evaluating coronary microcirculatory function, has been reported in only six papers. We believe this is mostly due to the importance of testing for individuals with coronary artery obstruction <50% is not sufficiently emphasized in Chinese clinical treatment. Therefore, more epidemiological investigations and prognostic studies are required to raise awareness among healthcare professionals about the need for early diagnosis and treatment of MVA. Thirdly, although we performed a subgroup analysis of the high heterogeneity, the results show that heterogeneity was significantly reduced in some subgroups. However, the meta-regression analysis suggests that there are still some factors that contribute to high heterogeneity, including acceptable differences across medical institutions, testers, and testing methods. Last but not least, in the analysis of publication bias, we found a significant publication bias for “hs-CRP,” which makes the conclusions regarding this indicator less credible. We speculate that this is due to the small quantity and inferior quality of the included literature.

## 5. Conclusion

The present study indicates that PCMs with conventional treatment of MVA could improve coronary microvascular function and clinical symptoms in MVA patients. Due to the limited number of literature studies and high heterogeneity, it is prudent to approach the conclusions with caution. In the future, we expectmore high-quality randomized double-blind clinical studies to validate the safety and efficacy of PCMs so as to provide more valuable evidence-based medical evidence for the treatment of MVA with PCMs.

## Figures and Tables

**Table 1:** Search strategy with PubMed as an example.

Search number	Query
#1	“Microvascular angina” [Mesh]
#2	(((((((((((Angina, microvascular) OR (X syndrome, angina)) OR (angina X syndrome)) OR (angina X syndromes)) OR (syndrome, angina X)) OR (syndrome X, cardiac)) OR (syndrome X, angina)) OR (angina syndrome X)) OR (angina syndrome Xs)) OR (syndrome Xs, angina)) OR (angina pectoris with normal coronary arteriogram)) OR (cardiac syndrome X)
#3	#1 OR #2
#4	“Medicine, Chinese traditional” [Mesh]
#5	((Zhong Yi Xue) OR (Chung I Hsueh)) OR (traditional Chinese medicine)
#6	#4 OR #5
#7	#3 AND #6
#8	((“Randomized controlled trial” (publication type)) OR (randomized (title/Abstract))) OR (clinical trials (title/Abstract))
#9	#7 AND #8

**Table 2:** Basic characteristics of included studies.

Study	Sample size	Age (years)	Sex M/F	Intervention measures		Treatment duration (days)	Outcomes	Adverse events
	*I*/*C*	*I*/*C*	*I*/*C*	*I*	*C*			
[14]	28/28	Not reported	Not reported	A + SBP 45 mg tid	A	42	(b)	None
[15]	60/60	*I*: 55.7 ± 3.2 *C*: 58.6 ± 4.4	*I*: 27/33 C:31/29	QSC 0.9 g tid	A	84	(b) + (c)	Upper abdominal discomfort
[16]	19/19	Not reported	Not reported	A+ TXLC 1.04 g tid	A	84	(b) + (d) + (e)	Not reported
[17]	34/32	Not reported	Not reported	A+ SBP 45 mg tid	A	180′	(c) + (d)	Not reported
[18]	37/33	*I*: 57 ± 15 *C*: 58 ± 13	*I*: 10/27 *C*: 9/24	A+ SBP 45 mg tid	A	180	(a)	None
[19]	35/30	*I*: 50.7 ± 6.3 |*C*: 51.3 ± 7.5	*I*: 19/16 *C*: 17/13	A+ SBP 45 mg tid	A	90	(c) + (d) + (e)	Not reported
[20]	34/34	*I*: 45.7 ± 6.9 *C*: 42.2 ± 6.0	*I*: 15/19 *C*: 16/18	A+ TXLC 0.78 g tid	A	180	(b) + (d) + (e)	Not reported
[21]	20/18	*I*: 51.00 ± 8.45 *C*: 51.95 ± 8.48	*I*: 7/13 *C*: 5/13	A+ STDP 70 mg tid	A	84	(b) + (c)	Not reported
[22]	30/32	*I*: 46.5 ± 3.3 *C*: 46.3 ± 3.2	*I*: 0/30 *C*: 0/32	A+ TXLC 1.04 g tid	A	90	(d) + (e)	Headaches
[23]	41/41	*I*: 53.35 ± 1.43 *C*: 53.13 ± 1.26	*I*: 14/27 *C*: 16/25	A+ YXTC 1.32 g tid	A	28	(b) + (d) + (e)	None
[24]	43/44	*I*: 57.3 ± 11.9 *C*: 56.1 ± 13.2	*I*: 23/20 *C*: 23/21	A+ YXSC 0.8 g tid	A	180	(a)+(c)+ (d)+(e)	Not reported
[25]	30/30	*I*: 48.93 ± 5.06 *C*: 47.77 ± 5.86	*I*: 7/23 *C*: 5/25	A+ XKSP 1.24 g tid	A	90	(c) + (d)	Not reported
[26]	38/39	*I*: 69.97 ± 8.48 *C*: 70.46 ± 7.75	*I*: 15/23 *C*: 18/21	A+ XKSP 1.24 g tid	A	180	(c)	Not reported
[27]	97/97	*I*: 63.0 ± 11.9 *C*: 65.0 ± 7.0	*I*: 23/20 *C*: 23/21	A+ QYDP 0.5 g tid	A	365	(a) + (b)	None
[28]	32/32	*I*: 41.38 ± 9.43 *C*: 45.75 ± 10.61	*I*: 18/14 *C*: 20/12	A+ SBP 45 mg tid	A	365	(a)	Not reported
[29]	54/52	*I*: 62.71 ± 5.32 *C*: 61.98 ± 5.39	*I*: 24/30 *C*: 23/29	A+ STDP 70 mg tid	A	84	(b) + (c) + (d) + (e)	Not reported
[30]	36/36	*I*: 61.23 ± 6.37 *C*: 60.92 ± 6.14	*I*: 18/18 *C*: 19/17	A+ STDP 70 mg tid	A	180	(a)	Not reported
[31]	34/34	*I*: 65.43 ± 4.29 C: 65.41 ± 4.27	*I*: 14/20 *C*: 15/19	A+ QSC 0.9 g tid	A	60	(d) + (e)	Headaches, dizziness, insomnia, nausea, vomiting, abdominal pain, palpitations
[32]	65/65	*I*: 57.82 ± 4.79 *C*: 58.17 ± 3.36	*I*: 28/37 *C*: 30/35	A+ YXLC 0.8 g tid	A	180	(d) + (e)	Palpitations diarrhea loss of appetite nausea and vomiting
[33]	20/20	*I*: 52.25 ± 10.14 *C*: 53.14 ± 9.10	*I*: 11/9 *C*: 12/8	A+ TXLC 1.04 g tid	A	180	(c)	None
[34]	39/39	*I*: 62.71 ± 7.24 *C*: 64.55 ± 6.14	*I*: 18/21 *C*: 20/19	A+ SBP 22.5 mg tid	A	90	(a) + (d) + (e)	Not reported

*I*, intervention group; *C*, control group; M, male; F, female; SBP, shexiang baoxin pill; QSC, qi shen capsule; TXLC, tong xin luo capsule; STDP, shexiang tongxin drop pill; YXSC, yindan xinnaotong soft capsule; YXTC, yu xin tong capsule; XKSP, xin ke shu pill; QYDP, qishen yiqi drop pill; A, conventional western medical treatment; (a) IMR; (b) TET; (c) hs-CRP; (d) ET-1; (e) NO.

**Table 3:** Subgroup analysis of IMR based on dosage form, sample size, treatment duration, sex ratio, average age, and location.

Items	Subgroup	*n*	SMD (95% CI)	*I * ^2^ (%)	*z*	*p*
Dosage form	Pill	3	−1.98 (−3.15, −0.82)	91.8	−3.327	0.001
	Dropping pill	2	−1.75 (−3.82, 0.32)	97.8	−1.655	0.098
	Capsule	1	−1.31 (−1.77, −0.84)	—	−5.515	<0.001

Sample size	≥80	2	−2.06 (−3.52, −0.59)	95.6	−2.756	0.006
	<80	4	−1.65 (−2.64, −0.66)	92.3	−3.276	0.001

Treatment duration	≥6 months	5	−1.51 (−2.29, −0.73)	92.5	−3.801	<0.001
	<6 months	1	−1.79 (−2.58, −1.00)	—	−9.373	<0.001

Sex ratio	F/M ≥ 100%	2	−2.42 (−4.05, −0.79)	92.9	−2.913	0.004
	F/M < 100%	4	−1.49 (−2.47, −0.51)	94.4	−2.981	0.003

Average age	>60	3	−2.24 (−3.79, −0.69)	96.5	−2.834	0.005
	<60	3	−1.34 (−1.63, −1.05)	0.0	−8.960	<0.001

Location	North	3	−2.39 (−3.61, −1.18)	93.8	−3.861	<0.001
	South	3	−1.19 (−1.71, −0.67)	70.4	−4.455	<0.001

**Table 4:** Subgroup analysis of TET based on dosage form, drug, sample size, treatment duration, sex ratio, average age, and location.

Items	Subgroup	*N*	SMD (95% CI)	*I * ^2^ (%)	*z*	*p*
Dosage form	Pill	1	0.24 (−0.28, 0.77)	—	0.907	0.364
	Dropping pill	3	0.58 (0.30, 0.85)	28.7	4.105	<0.001
	Capsule	4	2.68 (0.75, 4.60)	97.4	2.726	0.006

Drug	TXLC	2	0.76 (−0.59, 2.10)	90.4	1.105	0.269
	STDP	2	0.63 (0.12, 1.15)	49.5	2.402	0.016

Sample size	≥80	4	2.57 (1.01, 4.14)	97.9	3.217	0.001
	<80	4	0.52 (−0.12, 1.16)	79.3	1.595	0.111

Treatment duration	≥6 months	2	0.93 (0.01, 1.85)	89.2	1.982	0.047
	<6 months	6	1.74 (0.46, 3.01)	96.7	2.663	0.008

Sex ratio	F/M ≥ 100%	7	1.68 (0.60, 2.74)	96.1	3.065	0.002
	F/M < 100%	1	0.49 (0.20, 0.77)	—	3.349	0.001

Average age	>60	2	0.64 (0.29, 0.98)	50.6	3.634	<0.001
	<60	5	2.19 (0.59, 3.79)	97.0	2.678	0.007

Location	North	4	0.96 (0.15, 1.77)	93.8	2.327	0.020
	South	4	2.19 (0.04, 4.35)	97.4	1.922	0.046

**Table 5:** Subgroup analysis of hs-CRP based on dosage form, drug, sample size, treatment duration, sex ratio, average age, location, and year.

Items	Subgroup	*n*	SMD (95% CI)	*I * ^2^ (%)	z	*p*
Dosage form	Pill	4	−0.72 (−0.97, −0.48)	0.0	−5.712	<0.001
	Dropping pill	2	−2.49 (−5.16, 0.18)	96.9	−1.832	0.067
	Capsule	3	−1.00 (−1.92, −0.86)	90.4	−2.135	0.033

Drug	SBP	2	−0.79 (−1.16, −0.42)	6.6	−4.18	<0.001
	XKSP	2	−0.66 (−1.01, −0.32)	0.0	−3.77	<0.001

Sample size	≥80	3	−1.63 (−3.48, 0.22)	97.9	−1.729	0.084
	<80	6	−0.95 (−1.32, −0.59)	61.0	−5.111	<0.001

Treatment duration	≥6 months	4	−1.07 (−1.56, −0.57)	71.7	−4.219	<0.001
	<6 months	5	−1.29 (−2.43, −0.14)	95.9	−2.207	0.027

Sex ratio	F/M ≥ 100%	6	−1.41 (−2.42, −0.40)	95.3	−2.741	0.006
	F/M < 100%	3	−0.83 (−1.12, −0.56)	0.0	−5.911	<0.001

Average age	>60	2	−2.21 (−5.40, 0.98)	98.5	−1.358	0.175
	<60	7	−0.89 (−1.27–0.50)	74.6	−4.485	<0.001

Location	North	5	−1.26 (−2.34, −0.17)	95.9	−2.273	0.023
	South	4	−1.11 (−1.65, −0.57)	72.2	−4.038	<0.001

Year	2019	3	−0.76 (−1.03, −0.49)	0.0	−5.462	<0.001

**Table 6:** Subgroup analysis of ET-1 based on dosage form, drug, sample size, treatment duration, sex ratio, average age, location, and year.

Items	Subgroup	*n*	SMD (95% CI)	*I * ^2^ (%)	*z*	*p*
Dosage form	Pill	3	−1.70 (−2.16, −1.24)	52.0	−7.198	<0.001
	Dropping pill	1	−2.53 (−3.05, −2.02)	—	−9.670	<0.001
	Capsule	7	−1.68 (−2.13, −1.23)	79.6	−7.309	<0.001

Drug	TXLC	3	−1.73 (−2.67, −0.80)	84.7	−3.632	<0.001
	SBP	3	−1.70 (−2.16, −1.24)	52.0	−7.198	<0.001
	YXSC	2	−1.39 (−2.42, −0.36)	91.4	−2.636	0.008

Sample size	≥80	4	−1.83 (−2.53–1.13)	88.6	−5.109	<0.001
	<80	7	−1.73 (−2.11, −1.36)	65.5	−8.986	<0.001

Treatment duration	≥6 months	4	−1.63 (−2.20, −1.07)	80.5	−5.681	<0.001
	<6 months	7	−1.85 (−2.30, −1.41)	76.7	−8.143	<0.001

Sex ratio	F/M ≥ 100%	8	−1.85 (−2.20, −1.48)	73.0	−9.822	<0.001
	F/M < 100%	3	−1.56 (−2.34, −0.79)	84.0	−3.946	<0.001

Average age	>60	4	−1.88 (−2.37, −1.39)	74.9	−7.508	<0.001
	<60	7	−1.70 (−2.18, −1.22)	79.8	−6.936	<0.001

Location	North	8	−1.99 (−2.28, −1.70)	56.1	−13.529	<0.001
	South	3	−1.16 (−1.76, −0.56)	72.2	−3.774	<0.001

Year	2016	2	−1.82 (−2.22, −1.41)	0.0	−8.763	<0.001
	2018	2	−2.27 (−2.81, −1.74)	37.0	−8.259	<0.001

**Table 7:** Subgroup analysis of NO based-on dosage form, drug, sample size, treatment duration, sex ratio, average age, location, and year.

Items	Subgroup	*n*	SMD (95% CI)	*I * ^2^ (%)	*z*	*p*
Dosage form	Pill	3	1.11 (0.47, 1.76)	78.4	3.373	0.001
	Dropping pill	1	0.81 (0.42, 1.21)	—	4.017	<0.001
	Capsule	7	1.42 (0.95, 1.90)	82.9	5.900	<0.001

Drug	YXLC	3	1.03 (0.55, 1.51)	52.2	4.232	<0.001
	SBP	2	1.15 (0.05, 2.24)	89.2	2.052	0.040
	YXSC	2	1.18 (0.89, 1.46)	0.0	7.984	<0.001

Sample size	≥80	4	1.16 (0.87, 1.45)	45.4	7.872	<0.001
	<80	7	1.35 (0.78, 1.98)	86.4	4.611	<0.001

Treatment duration	≥6 months	3	1.05 (0.77, 1.33)	21.4	7.267	<0.001
	<6 months	8	1.39 (0.90, 1.87)	84.7	5.626	<0.001

Sex ratio	F/M ≥ 100%	9	1.37 (0.97, 1.76)	81.7	6.756	<0.001
	F/M < 100%	2	0.89 (0.32, 1.46)	64.7	3.068	0.002

Average age	>60	4	1.67 (0.87, 2.48)	91.3	4.062	<0.001
	<60	7	1.07 (0.79, 1.35)	49.5	7.510	<0.001

Location	North	7	1.39 (0.89, 1.89)	86.1	5.407	<0.001
	South	4	1.10 (0.73, 1.48)	51.2	5.819	<0.001

Year	2016	2	0.65 (0.30, 1.00)	0.0	3.655	<0.001
	2018	2	1.53 (1.16, 1.90)	0.0	8.042	<0.001
	2019	2	1.12 (0.77, 1.47)	0.0	6.295	<0.001

**Table 8:** Adverse events reported in various studies.

Study	Adverse events	Number of patients	
		*I*/*A*	*C*/*A*
[14]	Upper abdominal discomfort	1/60	0/60
[22]	Headaches	1/30	2/32
[31]	Headaches, dizziness, insomnia, nausea, vomiting, abdominal pain, and palpitations	5/34	3/34
[32]	Palpitations diarrhea loss of appetite nausea and vomiting	3/65	12/65
Total		10/189	17/191

*I*, occurrences in the intervention group; *C*, occurrences in the control group; *A*, Overall sample size of the study.

**Table 9:** Publication bias.

Indicators	Standard error	*t*	*p*	95% confidence interval
Efficacy assessment	4.4069	−1.52	0.171	(−17.1402, 3.701128)
Index of microvascular resistance	10.97181	−0.02	0.983	(−30.70549, 30.21979)
Time of duration on the treadmill exercise testing	4.53926	1.60	0.161	(−3.854731, 18.35961)
Hypersensitive C-reactive protein	4.127414	−2.74	0.029	(−21.07893, −1.559362)
Endothelin-1	4.316603	−0.78	0.454	(−13.14097, 6.388696)
Nitric oxide	3.36444	1.76	0.112	(−1.683471, 13.53831)

## Data Availability

The data supporting the findings of the study are available from the corresponding author upon request.

## Supplementary Materials

Supplementary Materials Supplementary data associated with this article can be found in the supplementary materials. Figure S1, S7, S14, S22, and S30 show the meta-analysis result of each item. Figure S2–S6, S8–S13, S15–S21, S23–S29, and S31–S37 show the subgroup analysis of factors that may lead to high heterogeneity. Table S1–S4 shows the results of the meta-regression of TET, hs-CRP, ET-1, and NO.
