# Supplementary material for: Effect of Proprietary Chinese Medicine on Coronary Microvascular Dysfunction in Patients with Microvascular Angina: A Systematic Review and Meta-Analysis
Source: Evid Based Complement Alternat Med. 2023 Jan 24;2023:9242752. doi: 10.1155/2023/9242752 (PMC9889144; doi:10.1155/2023/9242752)
Supplement: Supplementary Materials — Supplementary data associated with this article can be found in the supplementary materials. Figure S1, S7, S14, S22, and S30 show the meta-analysis result of each item. Figure S2–S6, S8–S13, S15–S21, S23–S29, and S31–S37 show the subgroup analysis of factors that may lead to high heterogeneity. Table S1–S4 shows the results of the meta-regression of TET, hs-CRP, ET-1, and NO. [file 9242752.f1.docx]

Supplementary Material

Effect of Proprietary Chinese Medicine on Coronary Microvascular Dysfunction in Patients with Microvascular Angina: A Systematic Review and Meta-analysis

Qiuyu Yu^1,†^, Xiaoyu Xu^1,†^, Shun Wang^1^, Yu Fan^1^, Jian Zhang^1^, Yingshu Leng^2^, Fuming Liu^1*^

^1^ Affiliated Hospital of Nanjing University of Chinese Medicine, Jiangsu Province Hospital of Chinese Medicine, First Clinical Medical College, Nanjing University of Chinese Medicine, Nanjing, Jiangsu, China.

^2^ Department of Biomedical Engineering, University of Ottawa, Ottawa, ON, K1N 6N5, Canada

^*^Correspondence author: Fuming Liu, The Affiliated Hospital of Nanjing University of Chinese Medicine, Nanjing, Jiangsu, China. Email: fsyy00652@njucm.edu.cn

^†^These authors contributed equally to this work and share first authorship

**Figure S1.** forest plot of IMR.

**Figure S2.** forest plot of IMR and subgroup analysis based on dosage form.

**Figure S3.** forest plot of IMR and subgroup analysis based on sample size.

**Figure S4.** forest plot of IMR and subgroup analysis based on treatment duration.

**Figure S5.** forest plot of IMR and subgroup analysis based on sex ratio.

**Figure S6.** forest plot of IMR and subgroup analysis based on location.

**Figure S7.** forest plot of TET.

**Figure S8.** forest plot of TET and subgroup analysis based on drug.

**Figure S9.** forest plot of TET and subgroup analysis based on sample size.

**Figure S10.** forest plot of TET and subgroup analysis based on treatment duration.

**Figure S11.** forest plot of TET and subgroup analysis based on sex ratio.

**Figure S12.** forest plot of TET and subgroup analysis based on average age.

**Figure S13.** forest plot of TET and subgroup analysis based on location.

**Figure S14.** forest plot of hs-CRP.

**Figure S15.** forest plot of hs-CRP and subgroup analysis based on dosage form.

**Figure S16.** forest plot of hs-CRP and subgroup analysis based on drug.

**Figure S17.** forest plot of hs-CRP and subgroup analysis based on sample size.

**Figure S18.** forest plot of hs-CRP and subgroup analysis based on treatment duration.

**Figure S19.** forest plot of hs-CRP and subgroup analysis based on sex ratio.

**Figure S20.** forest plot of hs-CRP and subgroup analysis based on average age.

**Figure S21.** forest plot of hs-CRP and subgroup analysis based on location.

**Figure S22.** forest plot of ET-1.

**Figure S23.** forest plot of ET-1 and subgroup analysis based on dosage form.

**Figure S24.** forest plot of ET-1 and subgroup analysis based on drug.

**Figure S25.** forest plot of ET-1 and subgroup analysis based on sample size.

**Figure S26.** forest plot of ET-1 and subgroup analysis based on treatment duration.

Figure S27. forest plot of ET-1 and subgroup analysis based on sex ratio.

**Figure S28.** forest plot of ET-1 and subgroup analysis based on average age.

**Figure S29.** forest plot of ET-1 and subgroup analysis based on location.

Figure S30. forest plot of NO.

Figure S31. forest plot of NO and subgroup analysis based on dosage form.

Figure S32. forest plot of NO and subgroup analysis based on drug.

Figure S33. forest plot of NO and subgroup analysis based on sample size.

**Figure S34.** forest plot of NO and subgroup analysis based on sex ratio.

**Figure S35.** forest plot of NO and subgroup analysis based on average age.

**Figure S36.** forest plot of NO and subgroup analysis based on location.

**Figure S37.** forest plot of NO and subgroup analysis based on location.

**Table S1.** Meta-regression of TET

**Table S2.** Meta-regression of hsCRP

**Table S3.** Meta-regression of ET-1

**Table S4.** Meta-regression of NO


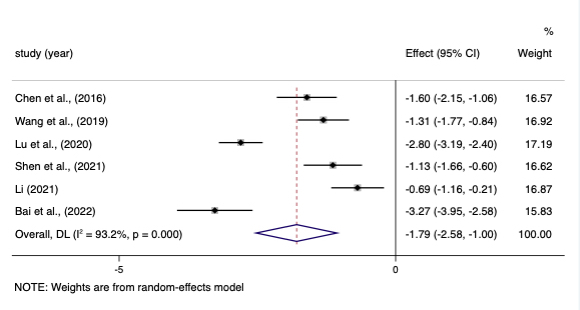


**Figure S1.** forest plot of IMR.


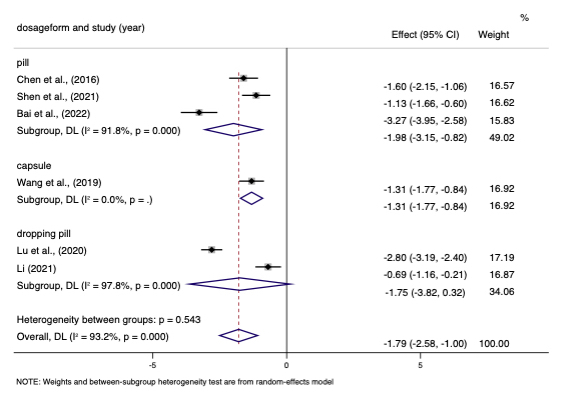


Figure S2. forest plot of IMR and subgroup analysis based on dosage form.


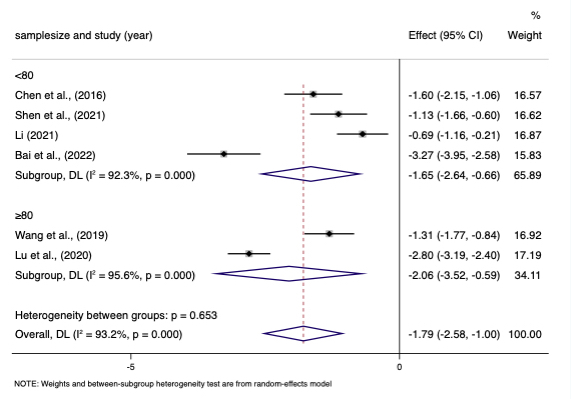


Figure S3. forest plot of IMR and subgroup analysis based on sample size.


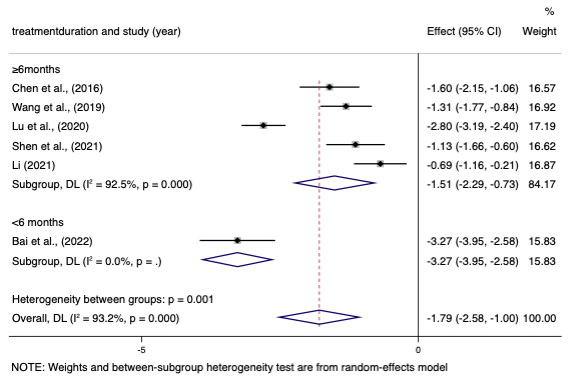


Figure S4. forest plot of IMR and subgroup analysis based on treatment duration.


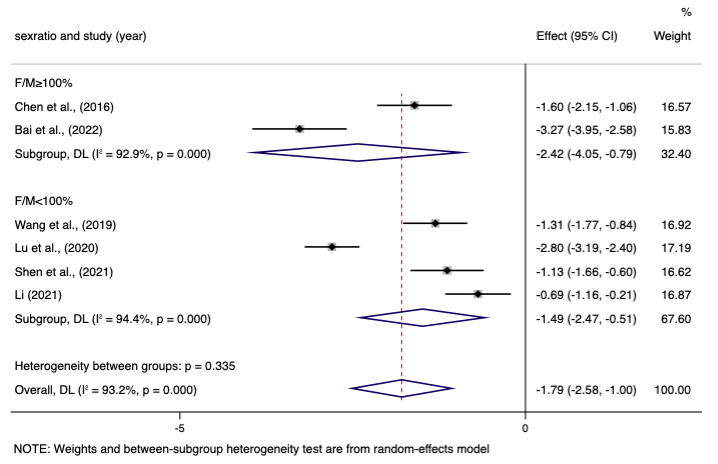


Figure S5. forest plot of IMR and subgroup analysis based on sex ratio.


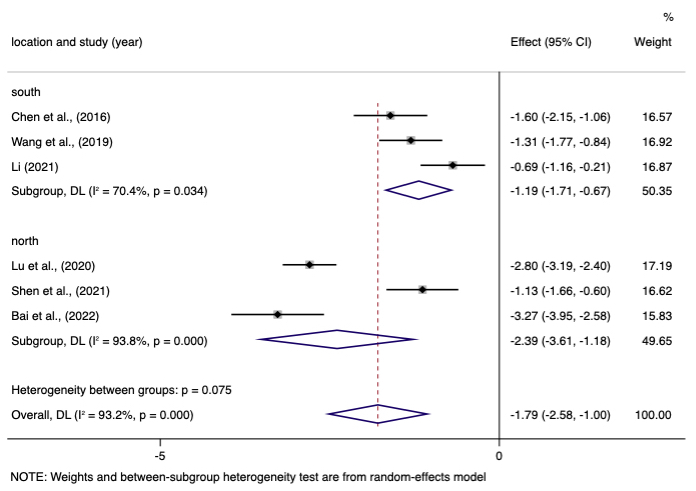


Figure S6. forest plot of IMR and subgroup analysis based on location.


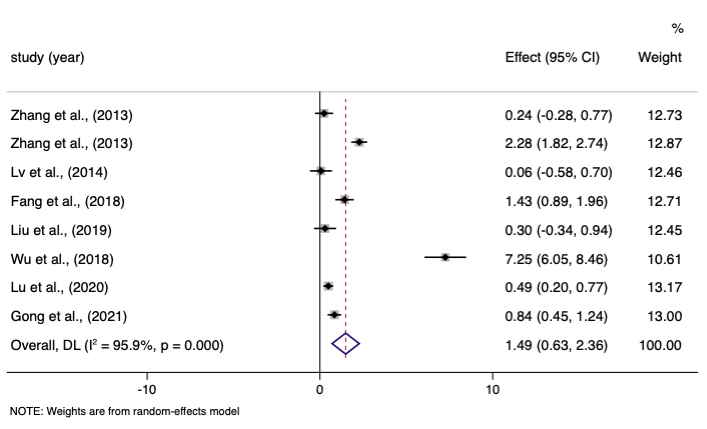


Figure S7. forest plot of TET.


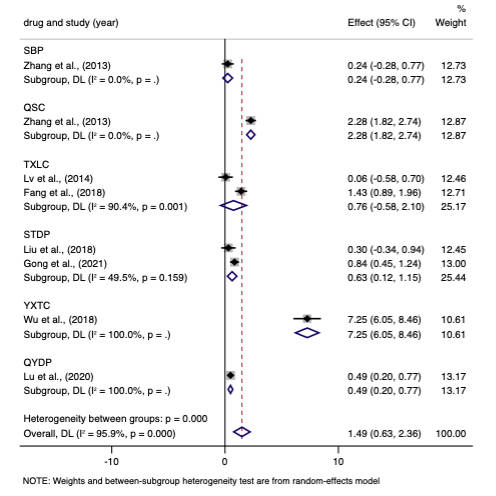


Figure S8. forest plot of TET and subgroup analysis based on drug.


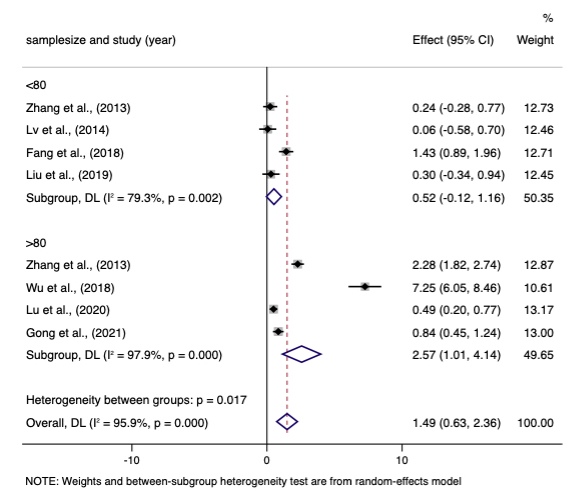


Figure S9. forest plot of TET and subgroup analysis based on sample size.


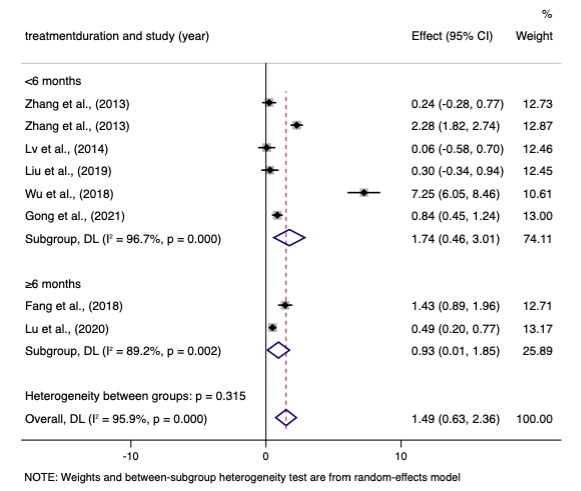


Figure S10. forest plot of TET and subgroup analysis based on treatment duration.


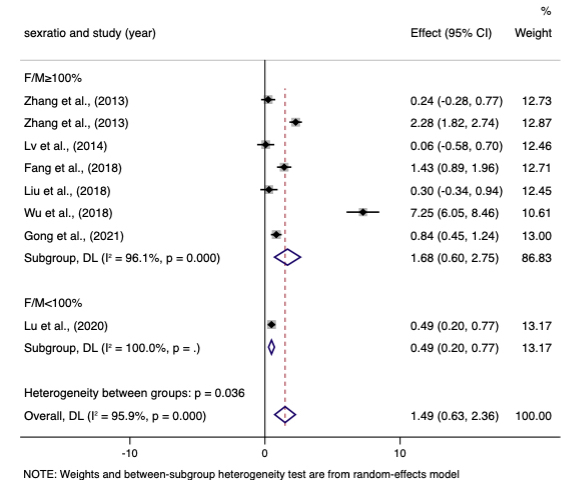


Figure S11. forest plot of TET and subgroup analysis based on average age.


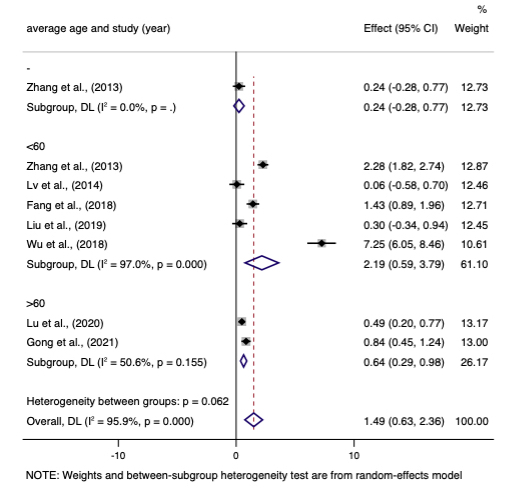


Figure S12. forest plot of TET and subgroup analysis based on average age.


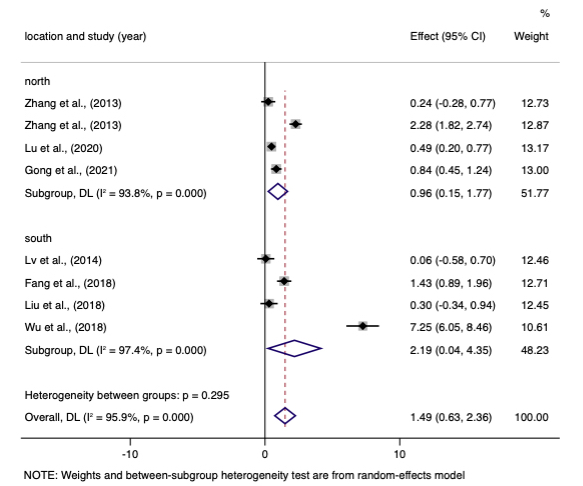


Figure S13. forest plot of TET and subgroup analysis based on location.


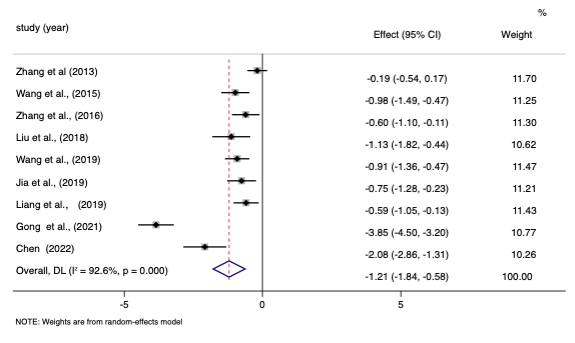


Figure S14. forest plot of hs-CRP.


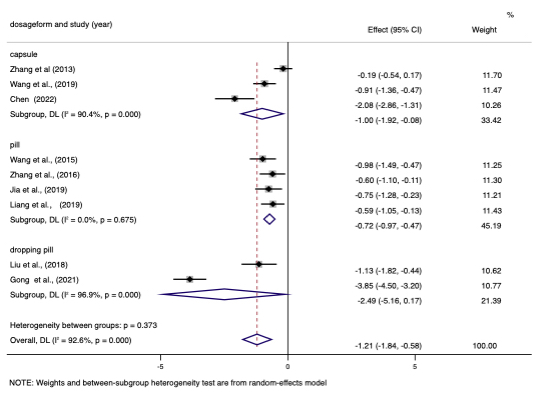


Figure S15. forest plot of hs-CRP and subgroup analysis based on dosage form.


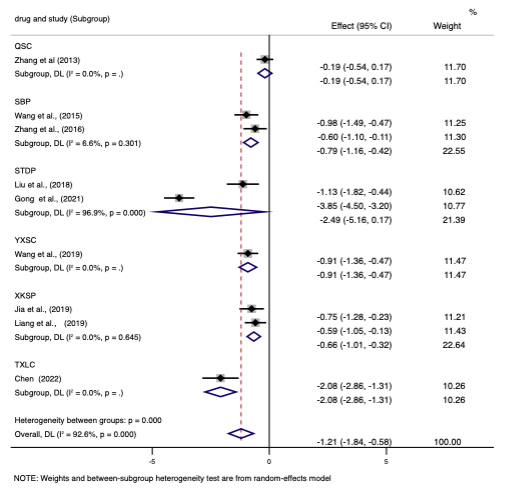


Figure S16. forest plot of hs-CRP and subgroup analysis based on drug.


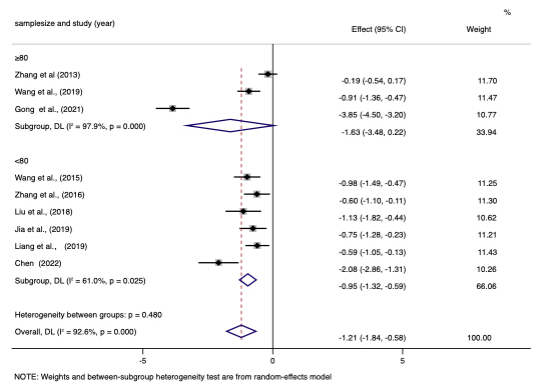


Figure S17. forest plot of hs-CRP and subgroup analysis based on sample size.


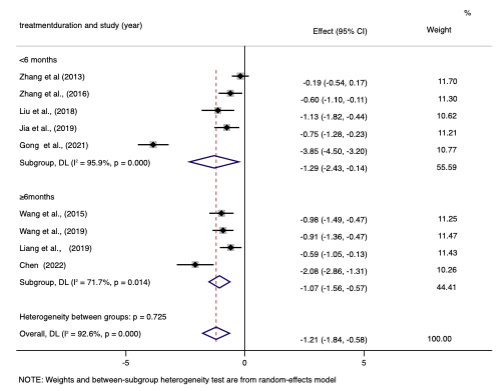


Figure S18. forest plot of hs-CRP and subgroup analysis based on treatment duration.


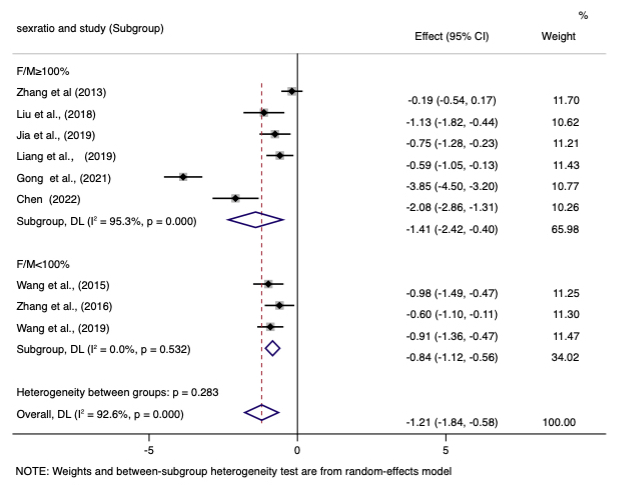


Figure S19. forest plot of hs-CRP and subgroup analysis based on sex ratio.


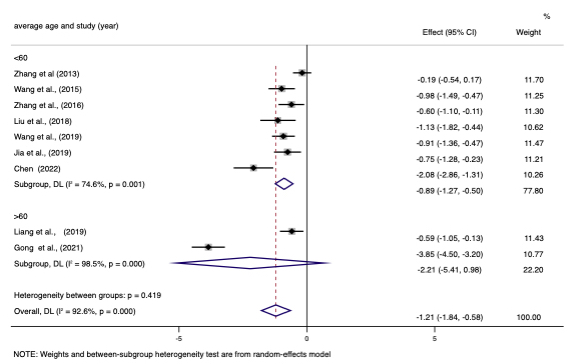


Figure S20. forest plot of hs-CRP and subgroup analysis based on average age.


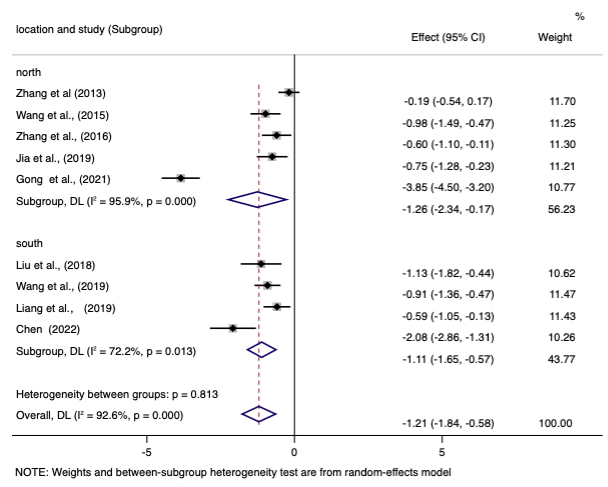


**Figure S21.** forest plot of hs-CRP and subgroup analysis based on location.


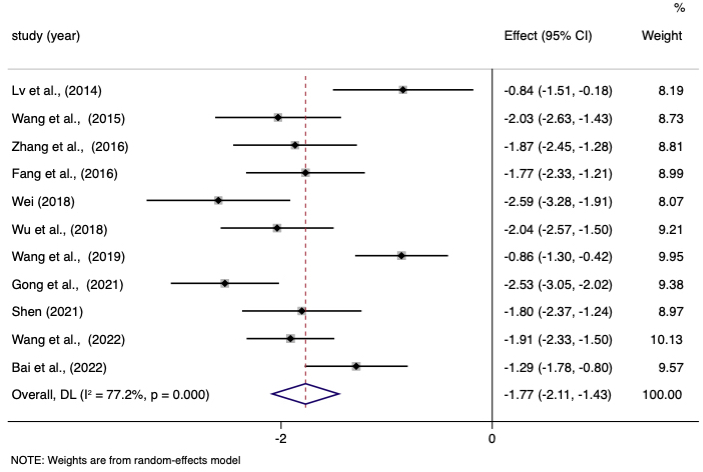


Figure S22. forest plot of ET-1.


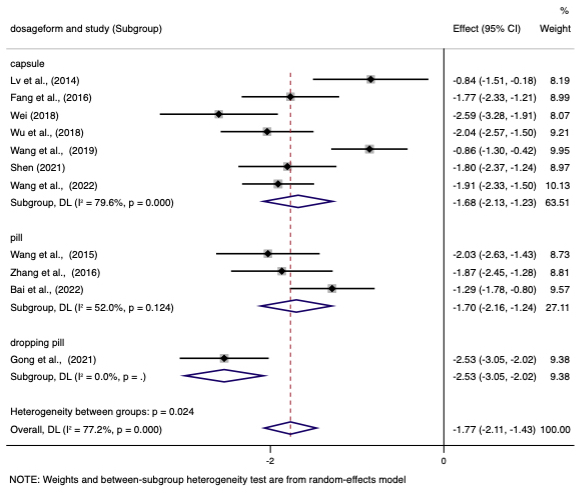


Figure S23. forest plot of ET-1 and subgroup analysis based on dosage form.


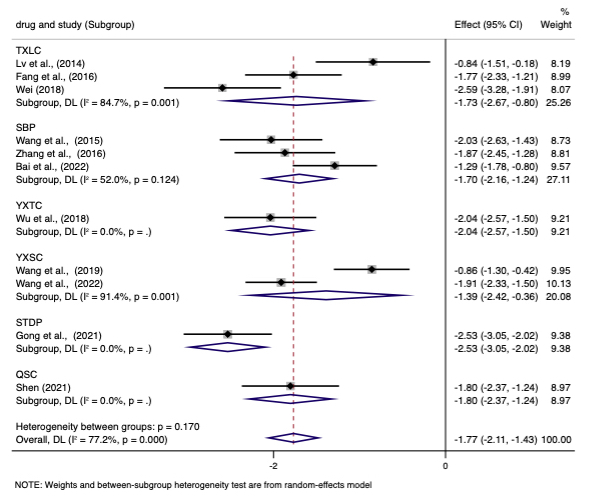


Figure S24. forest plot of ET-1 and subgroup analysis based on drug.


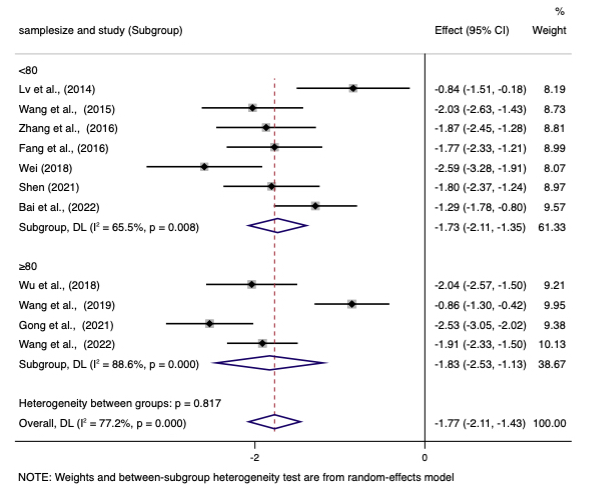


Figure S25. forest plot of ET-1 and subgroup analysis based on sample size.


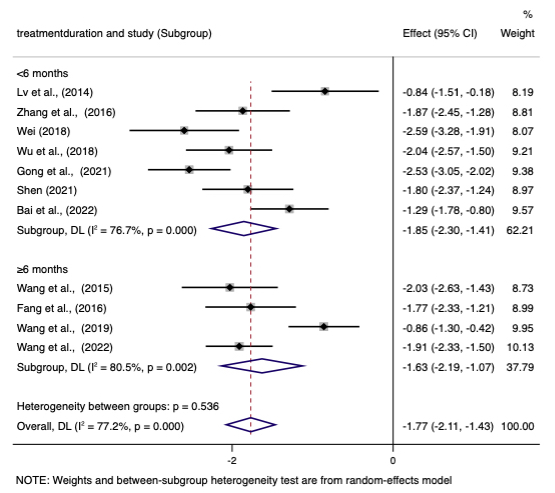


Figure S26. forest plot of ET-1 and subgroup analysis based on treatment duration.


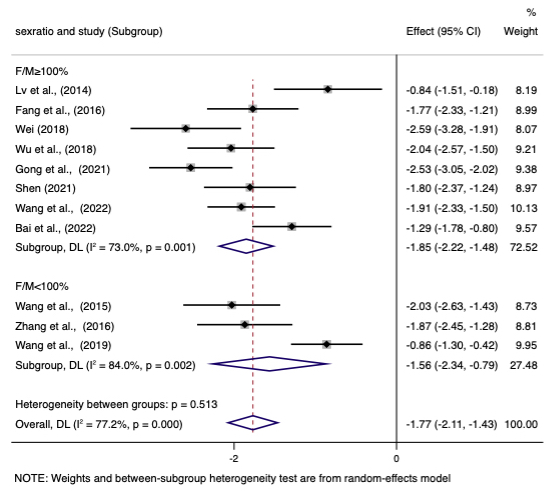


Figure S27. forest plot of ET-1 and subgroup analysis based on sex ratio.


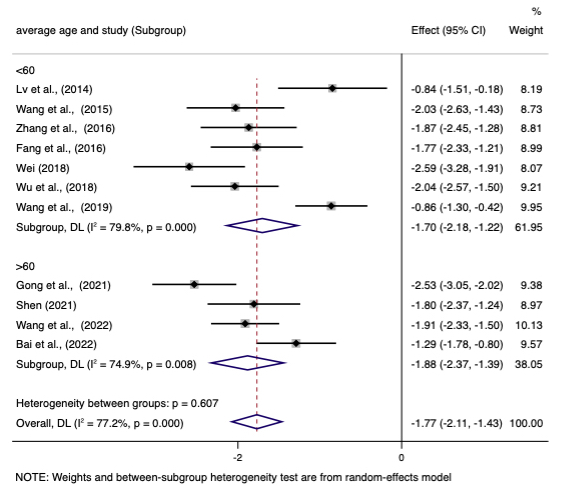


Figure S28. forest plot of ET-1 and subgroup analysis based on average age.


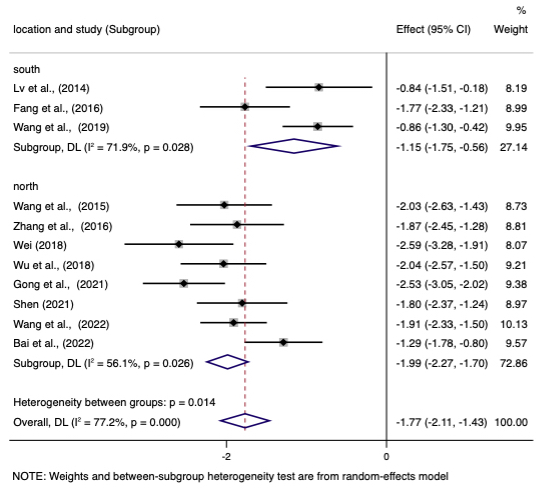


Figure S29. forest plot of ET-1 and subgroup analysis based on location.


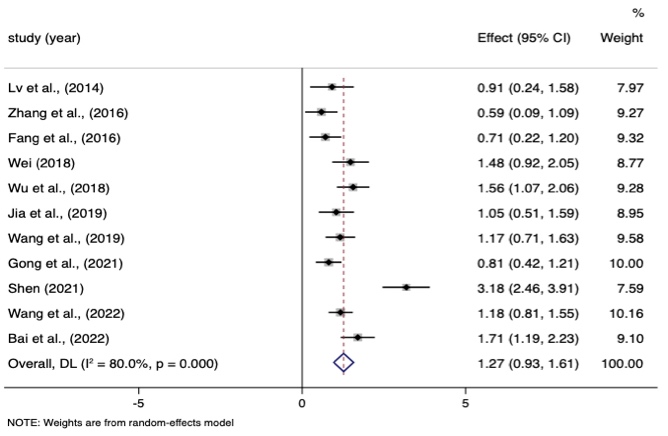


Figure S30. forest plot of NO.


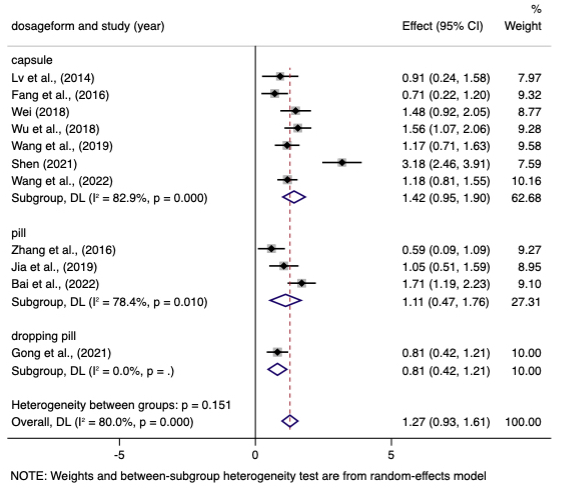


Figure S31. forest plot of NO and subgroup analysis based on dosage form.


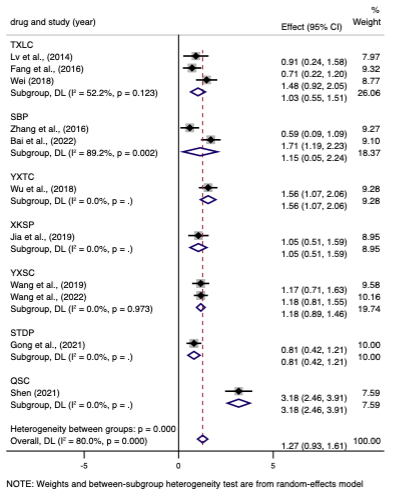


Figure S32. forest plot of NO and subgroup analysis based on drug.


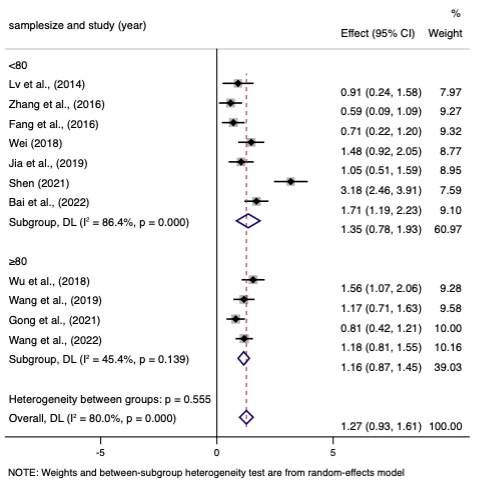


Figure S33. forest plot of NO and subgroup analysis based on sample size.


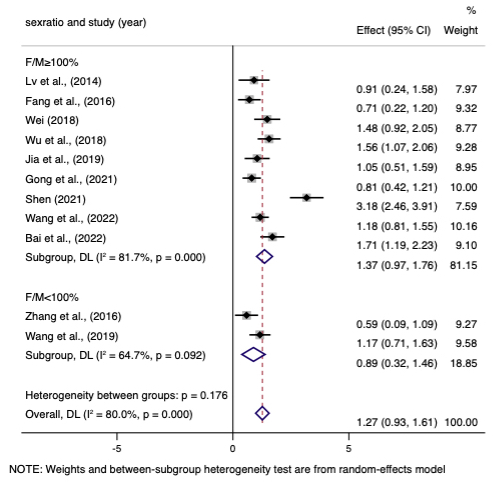


Figure S34. forest plot of NO and subgroup analysis based on sex ratio.


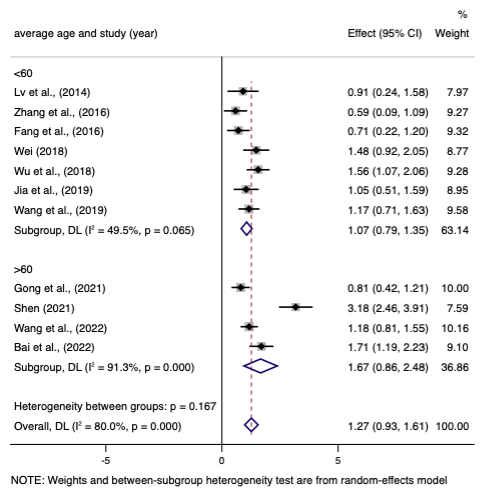


Figure S35. forest plot of NO and subgroup analysis based on average age.


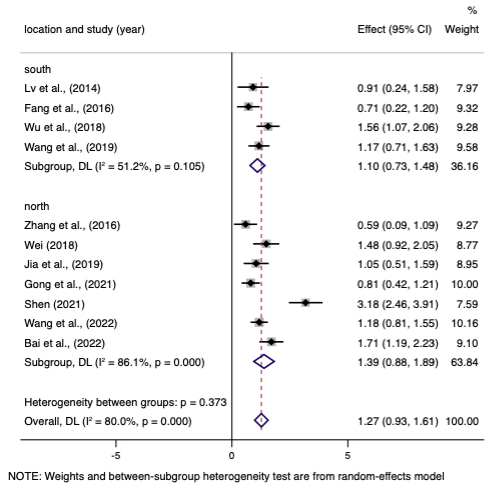


Figure S36. forest plot of NO and subgroup analysis based on location.


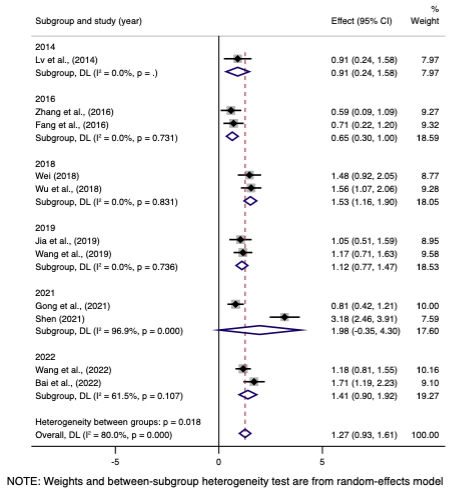


Figure S37. forest plot of NO and subgroup analysis based on location.

Table S1 Meta-regression of TET

| _ES | Standard Error | t | P | 95% Confidence Interval |
| --- | --- | --- | --- | --- |
| id1 | 1.801302 | -0.12 | 0.921 | (-23.11103，22.66439) |
| id2 | 1.650385 | 3.75 | 0.166 | (-14.78222，27.15803) |
| id3 | 1.792243 | 0.50 | 0.703 | (-21.87033，23.67487) |
| id4 | 2.430158 | -0.33 | 0.795 | (-31.68765，30.06851) |
| id5 | 2.453719 | -1.05 | 0.486 | (-33.74387，28.61105) |
| id6 | 1.265214 | 2.79 | 0.219 | (-12.54774，19.60441) |
| _cons | 4.395134 | -2.23 | 0.268 | (-65.64409，46.04684) |

Note: id1, dosage form; id2, sample size; id3, treatment duration; id4, sex ratio; id5, average age; id6, location. Statistical significance was determined at P < 0.05, indicating that the factor is associated with heterogeneity.

Table S2 Meta-regression of hsCRP

| _ES | Standard Error | t | P | 95% Confidence Interval |
| --- | --- | --- | --- | --- |
| year | 0.195949 | -1.54 | 0.368 | (-2.790553, 2.188982) |
| id1 | 0.9679343 | -0.89 | 0.537 | (-13.15988, 11.43766) |
| id2 | 1.103858 | 1.03 | 0.490 | (-12.88572, 15.16598) |
| id3 | 1.64684 | -0.57 | 0.672 | (-21.85702, 19.99314) |
| id4 | 1.253568 | 0.31 | 0.809 | (-15.5404, 16.31578) |
| id5 | 1.627422 | 0.36 | 0.782 | (-20.09895, 21.25775) |
| id6 | 1.198317 | 0.90 | 0.535 | (-14.15148, 16.30065) |
| _cons | 394.3595 | 1.53 | 0.368 | (-4406.759, 5614.866) |

Note: id1, dosage form; id2, sample size; id3, treatment duration; id4, sex ratio; id5, average age; id6, location. Statistical significance was determined at P < 0.05, indicating that the factor is associated with heterogeneity.

Table S3 Meta-regression of ET-1

| _ES | Standard Error | t | P | 95% Confidence Interval |
| --- | --- | --- | --- | --- |
| year | 0.1858454 | 0.38 | 0.728 | (-0.5203942, 0.6624919) |
| id1 | 0.3641018 | -1.42 | 0.252 | (-1.674273, 0.6431956) |
| id2 | 0.4610561 | 0.20 | 0.856 | (-12.88572, 15.16598) |
| id3 | 0.5807013 | -1.58 | 0.212 | (-2.767007, 0.9290949) |
| id4 | 0.5635784 | 1.23 | 0.306 | (-1.100059, 2.487058) |
| id5 | 1.005004 | 0.64 | 0.566 | (-2.551881, 3.844862) |
| id6 | 0.669915 | -2.10 | 0.126 | (-3.540632, 0.7233053) |
| _cons | 373.1003 | -0.38 | 0.728 | (-1330.123, 1044.62) |

Note: id1, dosage form; id2, sample size; id3, treatment duration; id4, sex ratio; id5, average age; id6, location. Statistical significance was determined at P < 0.05, indicating that the factor is associated with heterogeneity.

Table S4 Meta-regression of NO

| _ES | Standard Error | t | P | 95% Confidence Interval |
| --- | --- | --- | --- | --- |
| year | 0.0799083 | 2.24 | 0.154 | (-0.1645195, 0.5231156) |
| id1 | 0.1642902 | -4.36 | 0.049 | (-1.422411, -0.0086437) |
| id2 | 0.2679506 | -2.57 | 0.124 | (-1.841183, 0.4646132) |
| id3 | 0.2442492 | -4.01 | 0.057 | (-2.02984, 0.0719989) |
| id4 | 0.255805 | 0.69 | 0.559 | (-.9228876, 1.278393) |
| id5 | 0.3715677 | 0.79 | 0.510 | (-1.303715, 1.893738) |
| id6 | 0.2989348 | -2.00 | 0.184 | (-1.883719, 0.688706) |
| id7 | .0718933 | 2.00 | 0.183 | (-0.16524, 0.4534236) |
| _cons | 160.7178 | -2.22 | 0.156 | (-1049.053, 333.9727) |

Note: id1, dosage form; id2, sample size; id3, treatment duration; id4, sex ratio; id5, average age; id6, location; id7, drug. Statistical significance was determined at P < 0.05, indicating that the factor is associated with heterogeneity.
